# Molecular homology between canine spontaneous oral squamous cell carcinomas and human head-and-neck squamous cell carcinomas reveals disease drivers and therapeutic vulnerabilities

**DOI:** 10.1016/j.neo.2020.10.003

**Published:** 2020-11-02

**Authors:** Franco Guscetti, Sina Nassiri, Erin Beebe, Inês Rito Brandao, Ramona Graf, Enni Markkanen

**Affiliations:** a Institute of Veterinary Pathology, Vetsuisse Faculty, University of Zürich, Zürich, Switzerland; b Bioinformatics Core Facility, SIB Swiss Institute of Bioinformatics, Lausanne, Switzerland; c Institute of Veterinary Pharmacology and Toxicology, Vetsuisse Faculty, University of Zürich, Zürich, Switzerland

**Keywords:** Comparative oncology, Laser-capture microdissection, FFPE tissue, RNA sequencing, palbociclib, CDK4/CDK6

## Abstract

Spontaneously occurring canine oral squamous cell carcinomas (COSCC) are viewed as a useful model for human head and neck squamous cell carcinomas (HNSCC). To date however, the molecular basis of COSCC remains poorly understood. To identify changes pertinent to cancer cells in COSCC, we specifically analyzed tumor cells and matched normal epithelium from clinical formalin-fixed paraffin-embedded specimens using laser-capture-microdissection coupled with RNA-sequencing (RNAseq). Our results identify strong contributions of epithelial-to-mesenchymal transition (EMT), classical tumor-promoting (such as E2F, KRAS, MYC, mTORC1, and TGFB1 signaling) and immune-related pathways in the tumor epithelium of COSCC. Comparative analyses of COSCC with 43 paired tumor/normal HNSCC from The Cancer Genome Atlas revealed a high homology in transcriptional reprogramming, and identified processes associated with cell cycle progression, immune processes, and loss of cellular differentiation as likely central drivers of the disease. Similar to HNSCC, our analyses suggested a ZEB2-driven partial EMT in COSCC and identified selective upregulation of KRT14 and KRT17 in COSCC. Beyond homology in transcriptional signatures, we also found therapeutic vulnerabilities strongly conserved between the species: these included increased expression of PD-L1 and CTLA-4, coinciding with EMT and revealing the potential for immune checkpoint therapies, and overexpression of CDK4/6 that sensitized COSCC to treatment with palbociclib. In summary, our data significantly extend the current knowledge of molecular aberrations in COSCC and underline the potential of spontaneous COSCC as a model for HNSCC to interrogate therapeutic vulnerabilities and support translation of novel therapies from bench to bedside.

## Highlights

• Laser-capture microdissection (LCM) followed by RNAseq reveals detailed insights into COSCC and molecular homologies to HNSCC.
• Identification of CDK4/6 as therapeutic vulnerability in COSCC.
• This underlines the potential of spontaneous COSCC as a model for HNSCC to interrogate therapeutic vulnerabilities and support translation of novel therapies from bench to bedside.

## Introduction

Head and neck squamous cell carcinomas (HNSCC) are a heterogeneous group of epithelial tumors frequent in humans [1], [2], [3]. The main challenge of HNSCC lies in local invasion into bone and metastatic disease that lead to death of about 50% of patients with HNSCC. Recent developments in sequencing technologies coupled with state-of-the-art analytical methods have unveiled a plethora of mutations and deregulated pathways in HNSCC but understanding their impact on tumor development and survival necessitates the availability of accurate disease models. Many different disease models have been used thus far, all of them with significant drawbacks (e.g., [4], [5], [6]). Most notably, a failure to reflect the natural evolution of the tumor and its specific interactions with the native stroma implies that these models do not fully recapitulate the complexity of naturally occurring, spontaneous HNSCC, much of which can heavily influence the course of the disease.

Based on the closely related pathophysiology, spontaneously occurring cancers in the domestic dog are increasingly viewed as valuable models to promote understanding of cancer biology and identify novel potential therapeutic targets [7], [8], [9]. In particular, similar tumor types at similar locations between dogs and humans offer the possibility to overcome many of the limitations of xenograft or genetically modified rodent tumor models. This might apply for spontaneous canine oral squamous cell carcinomas (COSCC) that have been proposed as models for human HNSCC [10]. COSCC are the second most prevalent malignant oral neoplasm in dogs, and most often develop on the maxillar or mandibular gingiva at an average age of 9 years [11]. Nontonsillar COSCC display aggressive locally invasive behavior also frequently invading bone, but are slow to metastasize, which generally leads to a good prognosis following treatment, provided they are detected early and excised with sufficient surgical margins [12]. Local recurrence and invasion are the major problems, with destruction of tissue causing massive pain and dysfunction in affected patients [11]. The relatively high prevalence of COSCC, in conjunction with a large pet dog population, results in availability of samples. The shorter lifespan of dogs also offers the possibility to complete clinical studies in much shorter time than with human patients. Lastly, dogs often closely live with their owners, resulting in their exposure to similar environmental carcinogens. Therefore, analyzing molecular homologies between HNSCC and COSCC is considered a valuable approach to identify key events driving the disease and novel targets for pharmacological intervention. Finally, given the metabolic similarities that allow extrapolation of toxicity-related preclinical data from dogs to humans, spontaneous tumors in dogs could also serve as models to accelerate translation of novel therapeutic approaches from bench to bedside.

To date however, COSCC remain poorly understood on a molecular level. Currently, only one single study has analyzed 7 oral COSCC cases – 3 of these with matching normal tissue - using bulk RNAseq to describe expression changes occurring in these tumors [10]. Analysis of further patient cohorts including matched normal tissue samples is of utmost importance to validate these findings and further explore the molecular similarities and differences between COSCC and HNSCC. In this context, it is becoming increasingly clear that tumors are a very heterogeneous mixture of a variety of different cells. In addition to epithelial cancer cells, tumors harbor many different types of non-neoplastic cells and varying amounts of extracellular matrix. Traditionally, analysis of tumor samples has been performed in bulk, meaning that results reflect the mixture of all cells present, not differentiating between the epithelial cancer cells and the remaining non-neoplastic cells. Thus, this approach complicates the correct attribution of the observed gene expression changes either to the cancer cells or to the stromal cells. The importance of differentially analyzing tumor cells and the surrounding stroma has become increasingly evident recently, also for HNSCC (e.g. [13]). Hence, our group has established a workflow to isolate specific subpopulations of cells from archival formalin-fixed paraffin-embedded (FFPE) tissue sections by laser-capture microdissection (LCM) followed by RNAseq analysis [14], [15], [16], [17]. Using this approach, we set out to specifically isolate tumor cells and matched normal epithelial cells from 10 cases of COSCC in order to analyze the molecular underpinnings of COSCC and its resemblance with human HNSCC.

## Results

### Transcriptome profiling of laser-capture microdissected tumor and matched normal epithelium from clinical COSCC specimens

**Figure 1:**
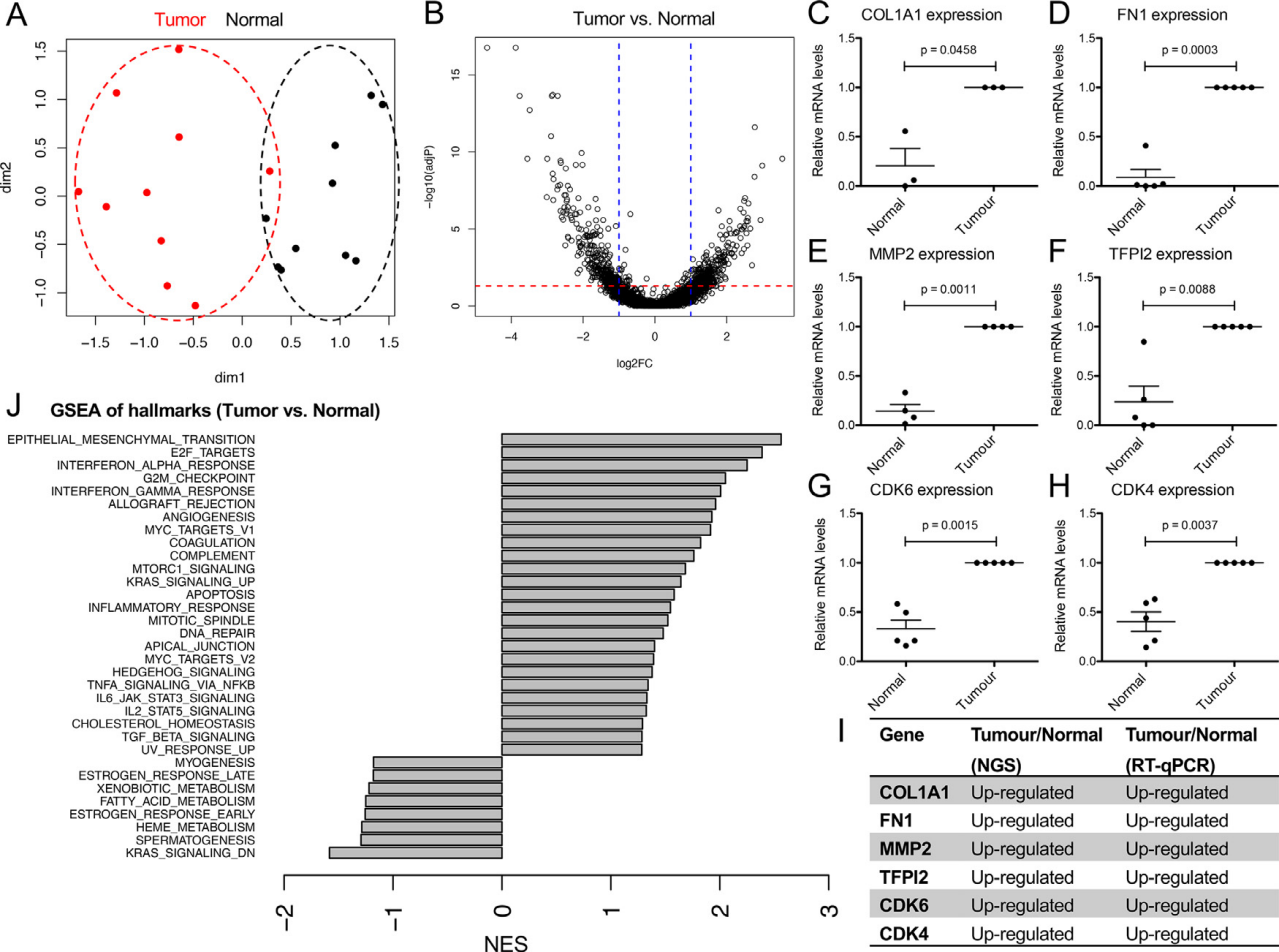
Transcriptome analysis of tumor cells and matched normal epithelium from 10 cases of canine oral squamous carcinoma. (A) Multidimensional scaling of tumor cells and normal epithelium isolated from canine oral squamous carcinoma using top 1000 variable genes. Each dot represents a sample, hence there are 20 dots in total (10 tumor and 10 normal), and distances between the dots in 2D approximate the log2 fold changes between the samples in multidimensional gene expression space. We used the top 1000 highly variable genes in the MDS analysis. (B) Volcano plot highlighting differentially expressed genes in tumor cells compared to normal epithelium, using |FC|>2 and FDR < 0.05 as cut-off values. (C–H): Expression levels of (C): COL1A1; (D): FN1; (E): MMP2; (F): TFPI2; (G): CDK6; (H): CDK4 as detected by qRT-PCR in normal or tumor epithelium, respectively. Values are mean values ±SEM, normalized to expression levels in tumor cells. *n* = 3–5 pairs. *P* values were calculated using student's *t* test with significance cutoff set at *P* = 0.05. I) Summary of the expression trends as detected by RNAseq and RT-qPCR. J) Gene-set enrichment analysis of hallmark gene sets deregulated between tumor and normal tissue. Hallmarks with FDR < 0.25 are shown. Positive normalized enrichment score (NES) indicates enrichment in tumor compared to normal tissue.

To analyze gene expression changes in COSCC, we specifically isolated tumor cells and matched normal epithelium from clinical FFPE specimens using LCM coupled with RNAseq as previously established [14], [15], [16], [17]. Representative images of tissue specimens and detailed patient characteristics for all cases are shown in Table 1 and Supplementary Figure 1. Unsupervised multidimensional scaling using the top 1000 variable genes showed separation of tumor samples from normal epithelium, suggesting clear differences in gene expression profiles between tumor and normal epithelium (Figure 1A). Indeed, differential expression analysis (FDR < 0.05 and fold change > 2) revealed 669 significantly differentially regulated genes between tumor and matched normal epithelium, with 340 up- and 329 down-regulated genes in tumor cells (Figure 1B and Supplementary Figure 1D). The full list of deregulated genes can be found in Supplementary Table 1. To validate the results from RNAseq, we measured expression of six genes (COL1A1, FN1, MMP2, TFPI2, CDK6, and CDK4) that were significantly up-regulated in tumor cells compared to normal epithelium by RT-qPCR. All of these genes showed significant expression changes consistent with RNAseq (Figure 1C–I). Taken together, these findings demonstrate the validity of specifically isolating tumor cells and matched normal epithelium from FFPE tissue sections and their analysis by RNAseq and reveal the occurrence of vast transcriptional reprogramming in COSCC.

**Table 1:** Overview of cases with COSCC included in this study.

Case no.	Age (y.)	Breed	Sex	Localization	Degree of Differentiation
1	5	Bolonka Zwetna	f/n	Oral cavity (gingiva)	Moderately differentiated
2	10	Collie	m	Oral cavity (gingiva)	Moderately differentiated
3	10	Poodle	m	Oral cavity (gingiva)	Moderately differentiated
4	6	American Cocker Spaniel	m	Oral cavity (tongue)	Moderately differentiated
5	14	West Highland White Terrier	m	Oral cavity	Well-differentiated
6	14	West Highland White Terrier	f/n	Oral cavity	Moderately differentiated
7	7	Long-Haired Collie	f/n	Oral cavity (gingiva)	Poorly differentiated
8	15	West Highland White Terrier	f/n	Oral cavity (gingiva)	Moderately differentiated
9	12	Cairn Terrier	m	Oral cavity (gingiva)	Moderately differentiated
10	10	n.d.	m	Oral cavity (gingiva)	Moderately differentiated

age = age at excision of tumour; f/n = female, neutered; m = male; n.d. = not disclosed.

### COSCC are characterized by epithelial-to-mesenchymal transition, cell cycle progression and activation of classical tumor-promoting and immune-related pathways

To understand the changes between tumor cells and normal epithelium, we performed gene set enrichment analysis (GSEA) of hallmark pathways obtained from the MSigDB database (http://software.broadinstitute.org/gsea). The following processes were significantly enriched (FDR < 0.25) in COSCC compared to normal epithelium: epithelial-to-mesenchymal transition (EMT), interferon alpha response, E2F targets, interferon gamma response, G2M checkpoint, angiogenesis, allograft rejection, coagulation, MYC targets, complement, KRAS signaling up, mTORC1 signaling, mitotic spindle, apoptosis, inflammatory response, STAT3 signaling, hedgehog signaling, STAT5 signaling, apical junction, DNA repair, TNFα signaling via NFκB, UV response up, and TGFβ signaling (Figure 1J). Processes that were significantly enriched (FDR < 0.25) in normal epithelium include: xenobiotic metabolism, fatty acid metabolism, hem metabolism, spermatogenesis, estrogen response early, and KRAS signaling down (Figure 1J). The clear emergence of an EMT signature was further supported by a strong down-regulation of the majority of detected keratins in tumor compared to normal epithelium, consistent with a loss of squamous cell differentiation (Table 2). The 2 exceptions to this are KRT14 and KRT17, both of which are heavily up-regulated in the tumor. Hence, COSCC cells display marked changes in EMT, cell cycle progression, immune-related responses and signaling pathways classically associated with tumor formation.

**Table 2:** Expression changes of selected Keratin genes pertaining to squamous differentiation.

Identifier	gene_name	log2 Ratio	*P* Value	FDR
ENSCAFG00000007208	KRT76	−6.469	1.04E-127	8.51E-124
ENSCAFG00000007233	KRT71	−5.807	3.05E-77	8.35E-74
ENSCAFG00000016017	KRT24	−5.62	1.48E-70	3.03E-67
ENSCAFG00000007322	KRT3	−3.363	6.91E-19	2.27E-16
ENSCAFG00000025402	KRT78	−3.173	2.47E-15	6.75E-13
ENSCAFG00000015999	KRT23	−2.824	3.87E-08	4.13E-06
ENSCAFG00000023449	KRT13	−2.816	2.37E-38	2.44E-35
ENSCAFG00000007328	KRT80	−2.662	1.62E-08	1.88E-06
ENSCAFG00000023529	KRT15	−1.929	2.71E-13	6.02E-11
ENSCAFG00000007204	KRT79	−1.798	0.001153	0.02762
ENSCAFG00000031250	KRT14	1.929	9.68E-08	9.03E-06
ENSCAFG00000007595	KRT17	3.767	8.02E-17	2.35E-14

Differentially regulated genes with a *P*-Value ≤ 0.01 and false-discovery rate (FDR) ≤ 0.1 were considered. Negative log2 Ratio values denote genes significantly down-regulated in tumor cells, while positive values are significantly up-regulated in tumor cells compared to the matched normal epithelium.

### High-grade homology in transcriptional reprogramming between COSCC and HNSCC identifies drivers of the disease

**Figure 2:**
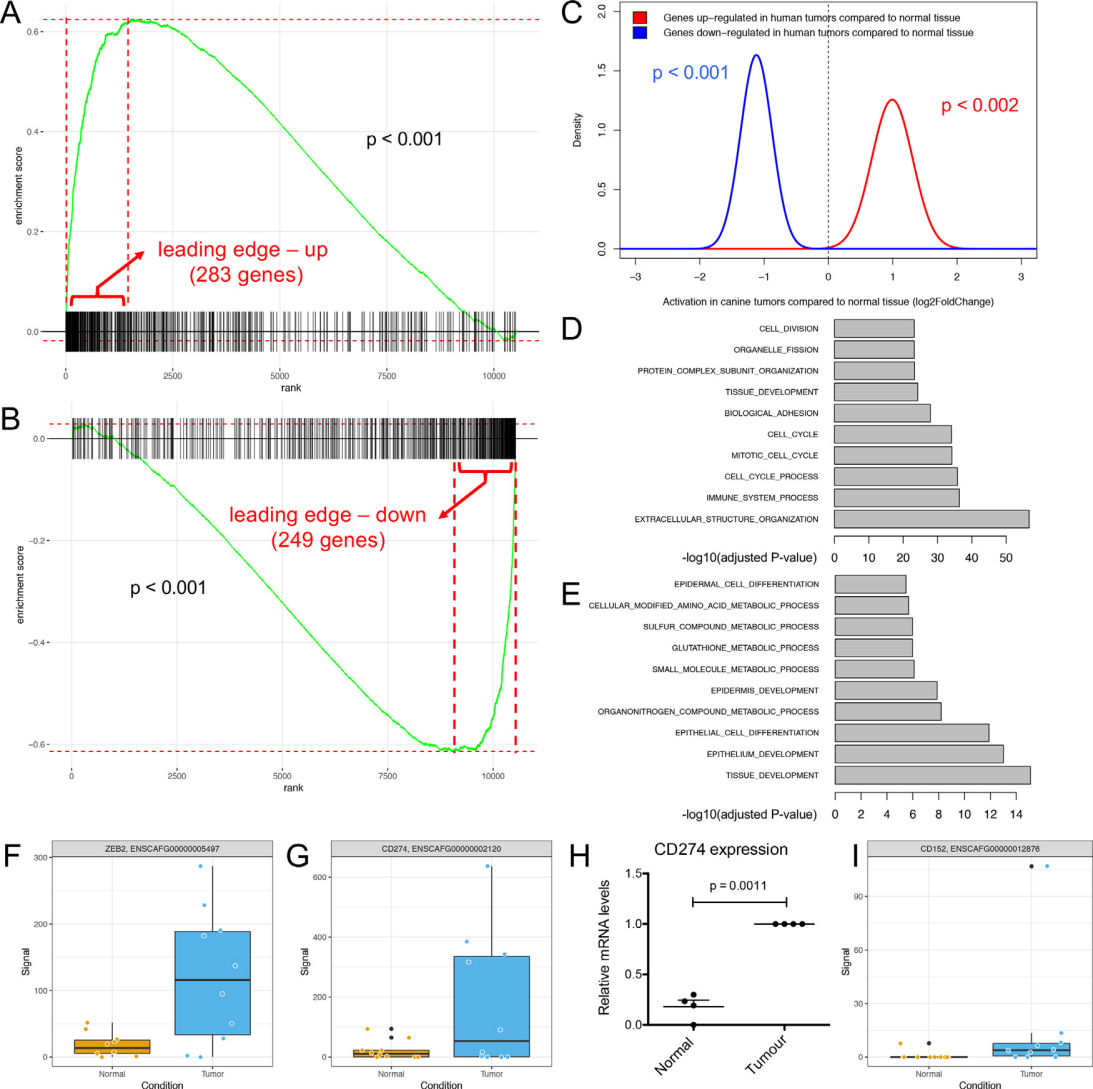
COSCC and HNSCC display a high grade of homology. (A and B) Competitive gene set testing to compare COSCC to HNSCC. GSEA-like running sum statistic depicting the location of (A) up-regulated and (B) down-regulated genes in HNSCC on a ranked list of genes in COSCC compared to normal. Permutation *P*-values were calculated by fgsea package. (C) Self-contained gene set testing (QuSAGE method) to assess the average differential expression of HNSCC gene sets (i.e., up- and down-regulated genes in HNSCC) in COSCC. X-axis demonstrates mean fold change expression in tumor compared to normal in COSCC (HNSCC data obtained from GSE62944). Y-axis indicates the distribution of fold change expression within each set. *P*-values were calculated by comparing mean fold change to fold change of 1 using Welch's *t*-test. (D-E) Hypergeometric *P*-values indicating enrichment of Gene Ontology biological processes (GObp) among the leading edge up-regulated (D) and down-regulated (E) genes as revealed by competitive gene testing in panels A and B. **(**F) Levels of ZEB2 in normal epithelium and tumor cells as detected by RNAseq. (G): Levels of CD274/PD-L1 as detected by RNAseq in normal and tumor epithelium, respectively. (H): Levels of CD274/PD-L1 as detected by qRT-PCR in normal and tumor epithelium, respectively. *P*-values were calculated using student's *t* test with significance cutoff set at *P* = 0.05. (I): Levels of CD152/CTLA-4 as detected by RNAseq in normal and tumor epithelium, respectively. COSCC, canine oral squamous cell carcinomas; HNSCC, head and neck squamous cell carcinomas.

To date there is only very limited data with respect to transcriptional reprogramming in COSCC, which strongly limits our understanding regarding the extent of molecular homology and difference between COSCC and HNSCC. To perform an unbiased comparative analysis of expression changes between HNSCC and COSCC, we made use of the TCGA data for HNSCC. Since our analysis of COSCC was based on comparing matched tumor/normal samples, we aimed for the same setup in the HNSCC data. One dataset (GSE62944) matched the criteria containing paired tumor/normal samples for all patients of tumors from different anatomical sites of the oral cavity (Supplementary Figure 2). We hypothesized that if there was molecular homology in transcriptional reprogramming of the tumor cells between the 2 species, up-regulated genes in human tumors should on average also show up-regulation in canine tumors. Similarly, down-regulated genes in human tumors should on average also demonstrate down-regulation in canine tumors. We tested this hypothesis using competitive (GSEA-like, Figure 2A and B) and self-contained (QuSAGE, Figure 2C) gene set testing. Reassuringly, we found strong agreement in the differential expression profiles between the 2 datasets, suggesting high-grade homology in transcriptional reprogramming between HNSCC and COSCC.

To further explore the genes whose expression was highly correlated between HNSCC and COSCC, we analyzed the leading edge of the up- and down-regulated genes (i.e., the genes that were highly similarly deregulated in both species) derived from GSEA analysis. The up-regulated leading edge consisted of 283, and the down-regulated leading edge of 249 genes (Figures 2A and B, and Supplementary Tables 2 and 3). Over-representation analysis of Gene Ontology biological processes among the leading edge up-regulated genes (as determined in Figure 2A) revealed the majority of genes to be involved in processes associated with cell cycle and division, immune processes, and extracellular restructuring (Figure 2D). Examination of the genes belonging to the down-regulated leading edge revealed the majority of these genes to be involved in processes associated with epithelial cell and tissue differentiation and development, and metabolic processes (Figure 2E). Interestingly, expression of one of the central EMT drivers, ZEB2, was significantly up-regulated in tumors compared to normal epithelium (Figure 2F; *P*-value 0.0065), while expression of other typical EMT regulators such as ZEB1, SNAI1, SNAI2, SNAI3 and TWIST2 either did not change, or could not be detected at a sufficient level (Supplementary Table 1). This very specific up-regulation of ZEB2 might suggest the presence of a partial EMT program in COSCC. Previous studies have demonstrated a significant association between EMT and expression of PD-L1 (CD274), one of the key molecules that block anti-tumor immune response, in HNSCC [22]. Accordingly, RNAseq revealed a significant increase in CD274 in canine tumor cells (Log2 fold-change = 1.527, *P*-value = 0.001), which was validated by qRT-PCR (Figures 2G and H). Moreover, expression of the immune inhibitory checkpoint receptor CTLA-4 (CD152) was significantly increased in tumor compared to normal epithelium (Log2 fold-change = 0.7106, *P*-value = 0.013) (Figure 2I). Thus, there is an increased expression of the immune inhibitory molecules PD-L1 and CTLA-4 in COSCC tumor cells that also display active EMT signaling, suggesting that COSCC patients could potentially benefit from immune checkpoint inhibitor therapies.

Taken together, these findings reveal a significant overlap between HNSCC and COSCC both in terms of deregulated genes and associated biological pathways, supporting the presence of extensive molecular homology between COSCC and HNSCC. As such, these data identify a partial EMT program and cell cycle progression as conserved central drivers of the disease in COSCC and reveal the potential for immune checkpoint inhibitor therapies for treatment of COSCC.

### Identification of CDK4/6 inhibition as therapeutic vulnerability of COSCC

**Figure 3:**
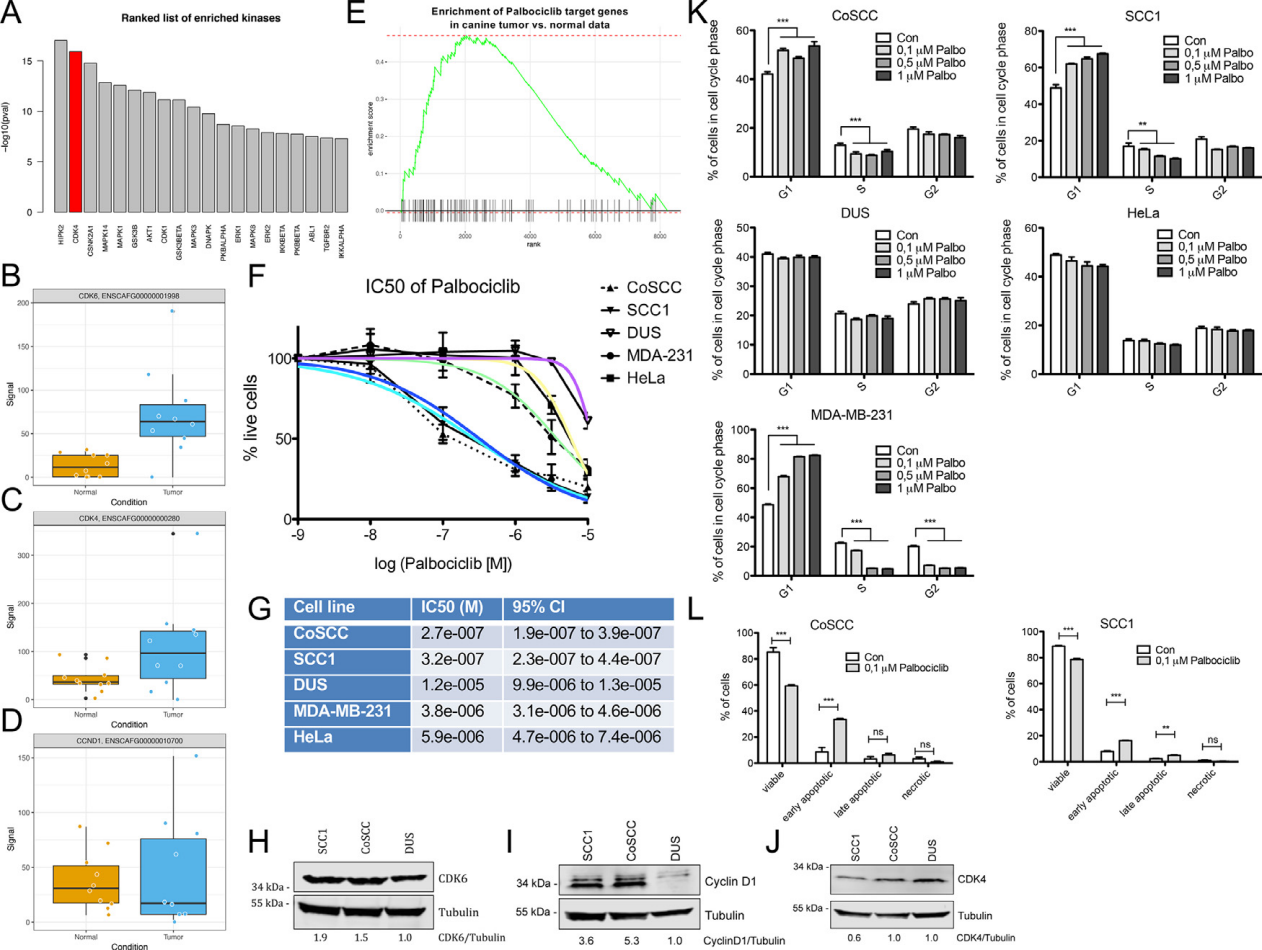
Identification of CDK4/6 inhibition by palbociclib as therapeutic vulnerability of COSCC. **(**A) Top 20 upstream kinases deduced from COSCC gene expression data using Expression2Kinases (X2K). CDK4 is the second most enriched kinase revealed by X2K. **(**B-D) Levels of CDK6 (B), CDK4 (C) and Cyclin D1 (D) mRNA in normal epithelium and tumor cells as detected by RNAseq. (E) GSEA analysis of palbociclib target genes in COSCC compared to normal epithelium (GSEA *P*-value < 0.001). Palbociclib target genes were obtained from the Comparative Toxicogenomics Database (CTD). (F) Sensitivity of cell lines to palbociclib treatment. The canine oral squamous cell carcinoma cell lines CoSCC and SCC1, and a canine stromal cell line DUS, as well as human HeLa and MDA-MB-231 cells were exposed to increasing concentrations of palbociclib for 72 h, after which the number of surviving cells was assessed using the Resazurin assay. The percentage of surviving cells was calculated in relation to control cells exposed to DMSO only. Data shown are mean from *n* = 6 independent assays ±SD. The colored lines indicate nonlinear fit IC50 curves for each cell line; purple: DUS, yellow: HeLa, green: MDA-231, dark blue: SCC1, light blue: CoSCC. **(**G) IC50 values of the cell lines tested in (F). Shown are IC50 and the 95% confidence intervals, as calculated using nonlinear regression (curve fit; normalized response with variable slope) from *n* = 6 independent assays with 4–8 replicates per data point. **(**H-J) Western blot of CDK6 (H), Cyclin D1 (I), and CDK4 (J) levels in the canine cell lines SCC1, CoSCC, and DUS. Tubulin served as loading control. Relative levels of CDK6, Cyclin D1, and CDK4 to tubulin, normalized to levels in DUS cells are indicated below the blots. **(**K) Cell cycle analysis of SCC1, CoSCC, DUS, HeLa and MDA-MB-231 cells after a 24 h treatment with palbociclib. Data shown are mean from *n* = 6 independent assays ±SEM. Significance was analyzed using Student's two-tailed *t* test; ** = *P* < 0.01, *** = *P* < 0.001. **(**L) Analysis of viable, early and late apoptotic and necrotic CoSCC and SCC1 after a 72 h treatment with palbociclib. Data shown are mean from *n* = 4 independent assays ±SEM. Significance was analyzed using Student's two-tailed *t* test; * = *P* < 0.05, ** = *P* < 0.01, *** = *P* < 0.001. COSCC, canine oral squamous cell carcinomas; HNSCC, head and neck squamous cell carcinomas; ns = not significant.

There is great interest in identification of therapeutic vulnerabilities in both HNSCC and COSCC that could be used to complement and/or reduce the extent of surgical intervention. To explore this avenue, we took advantage of the Expression2Kinases (X2K, [24]) computational framework to shed light on the upstream regulators likely responsible for observed patterns in the COSCC gene expression data. X2K aims to identify candidate genes that are likely responsible for observed changes in mRNA expression, which could be exploited as therapeutic targets. Figure 3A shows the top kinases upstream of differentially expressed genes identified in COSCC compared to normal epithelium as revealed by X2K. The second hit, CDK4 caught our attention as inhibitors of CDK4/6 are readily available and already in clinical use [25]. An involvement of CDK4 was in agreement with the strong enrichment of E2F signaling as revealed by GSEA (Figure 1J), a regulator of cell cycle progression via phosphorylation of RB by cyclin D‐CDK4/6. Given our findings on CDK4/6 and E2F signaling, we analyzed the levels of CDK4, CDK6 and Cyclin D1 in COSCC. Indeed, cyclin dependent kinase 6 (CDK6) was significantly overexpressed in tumor cells compared to normal epithelium (log2 fold change = 1.854, *P*-value = 0.007, Figure 3B), which was validated by RT-qPCR (Figures 1G and I). Similarly, qRT-PCR validation showed significantly elevated CDK4 in tumor cells (Figures 1H and I), albeit not significant in RNAseq (Log2 fold change = 0.9817, *P*-value = 0.115, Figure 3C). Cyclin D1 expression did not significantly differ between normal and tumor cells in RNAseq (Figure 3D). Both CDK4 and CDK6 can be pharmacologically inhibited by clinically approved inhibitors, such as palbociclib, in humans. Importantly, canine and human CDK4 and CDK6 are highly conserved, suggesting that palbociclib could be used to inhibit activity of CDK4/6 also in canine tumors. This hypothesis was further supported by the enrichment of genes encoding for proteins that interact with palbociclib, in COSCC compared to normal epithelium (Figure 3E, GSEA *P*-value < 0.001).

Inspired by these findings, we set out to investigate whether COSCC cells were sensitive to palbociclib in vitro. To assess this, 2 cancer cell lines directly derived from independent cases of COSCC (termed SCC1 and CoSCC, respectively), and a canine stromal cell line that served as nonepithelial control (termed DUS) were exposed to different concentrations of palbociclib in vitro. Human HeLa cells, known to have a high IC50 for palbociclib, and human MDA-MB-231 cells, known for a lower IC50, served as negative and positive controls, respectively [28]. As expected, HeLa cells showed a markedly higher IC50 than MDA-MB-231 cells (Figures 3F and G). Interestingly, both COSCC cell lines were highly sensitive to palbociclib, whereas growth of the canine stromal cell line DUS was hardly inhibited (Figures 3F and G). Of note, sensitivity of the 2 COSCC cell lines markedly exceeded that of the human MDS-MB-231 cells. Western blot analysis confirmed elevated levels of CDK6 and Cyclin D1 in both SCC1 and CoSCC compared to DUS cells, while CDK4 levels were comparable in all 3 cell lines (Figure 3H–J).

As CDK4/6 are responsible for the G1/S transition of the cell cycle, palbociclib treatment is expected to increase the number of cells in G1. Accordingly, cell cycle analysis of cells treated with concentrations ranging from 0.1 to 1 µM palbociclib for 24 h revealed a significant increase in the G1 cell population and a concomitant decrease in S-phase cells in the sensitive SCC1 and CoSCC and MDA-MB-231 cells (Figure 3K). In contrast, the cell cycle of DUS and HeLa cells did not alter upon palbociclib treatment, further corroborating the relative insensitivity of these 2 cell lines against palbociclib (Figure 3K). These findings suggest that the sensitivity of COSCC cells towards palbociclib is at least partially mediated through induction of a cell cycle arrest. Palbociclib has also been shown to induce apoptosis in sensitive cells. To understand whether an increase in apoptosis could also contribute to the strong sensitivity of COSCC cells towards palbociclib, we measured the rate of early and late apoptotic cells after exposure to 0.1 µM palbociclib for 72 h. Clearly, palbociclib induced a significant increase in the number of cells both in early and/or late apoptosis, and a concomitant decrease in viable cells in CoSCC and SCC1, suggesting that the sensitivity of COSCC cells towards palbociclib is driven by a combination of G1 arrest and apoptosis (Figure 3L). Importantly, as only partially viable cells that remain attached to the plates at the time of harvesting can be analyzed, these numbers are likely to substantially underestimate the extent of late apoptosis. In summary, our results demonstrate that COSCC overexpress CDK6 and/or CDK4, which renders them sensitive to induction of apoptosis by low doses of palbociclib. Thus, we have identified CDK4/6 overexpression as a potential therapeutic vulnerability in COSCC that should be further interrogated in a clinical setting. Given the current clinical trials addressing palbociclib and other CDK4/6 inhibitors for the treatment of HNSCC, these data strongly advocate that homology between COSCC and HNSCC extends far beyond similarities in transcriptional signatures, and allows interrogation of therapeutic vulnerabilities using COSCC as a model for HNSCC, to support translation of novel therapies from bench to bedside.

## Discussion

Here, we present a thorough transcriptome analysis of tumor cells and matched normal epithelium isolated from 10 cases of COSCC using LCM of FFPE tissue coupled with RNAseq. Our data provide detailed insight into transcriptional reprogramming of COSCC and identifies strong molecular homologies and therapeutic vulnerabilities shared between COSCC and HNSCC. As such, our findings validate and significantly extend an earlier analysis that suggested the existence of molecular homologies between COSCC and HNSCC [10] that extend far beyond similarities in transcriptional signature, provide information about clinically actionable targets for the treatment of COSCC, and advocate the interrogation of therapeutic vulnerabilities using COSCC as a model for HNSCC to support translation of novel therapies from bench to bedside.

We found wide-ranging molecular homologies between HNSCC and COSCC (Figure 2). Strongest overlaps among the up-regulated genes were found in processes associated with cell cycle and division, immune processes, and extracellular restructuring (Figure 2D), whereas overlaps among the down-regulated genes centered on epithelial cell and tissue differentiation and development, and metabolic processes, suggesting activation of an EMT program in COSCC (Figure 2E). This was further supported by gene set enrichment analysis of COSCC revealing a strong EMT signature (Figure 1J). EMT is a salient feature of HNSCC that has been recently identified in humans and strongly correlated with a more malignant tumor phenotype [13]. Further support for the activation of EMT derives from the fact that the majority of all detected keratins in tumor cells were down-regulated compared to normal epithelium, consistent with a loss of differentiation (Table 2). In strong contrast to this, KRT14 and KRT17 are both heavily up-regulated in COSCC. These 2 are among the top up-regulated keratins in HNSCC and other human SCC [13,[18], [19], [20]]. Expression of both KRT14 and KRT17 is associated with basal epithelial layers [29], and it has been suggested that high levels of KRT14 and KRT17 are needed to sustain a “stem-like” proliferative epidermal cell phenotype [30]. Finally, human squamous cell carcinomas are characterized by the expression of KRT14 and KRT17 [18]. Substantial EMT has also been suggested to occur in COSCC [10]. In the context of the present study, expression of one of the central EMT drivers, ZEB2, was also significantly up-regulated, while we did not detect changes in other typical EMT regulators. This suggests the presence of a partial ZEB2-orchestrated EMT program in COSCC, similarly to the SNAI2-driven partial EMT in HNSCC, which is clearly distinct from full EMT programs or “mesenchymal” tumor signatures derived from bulk tumor sequencing [13]. The loss of genes related to cell and tissue differentiation is in direct concordance with the observed loss of squamous cell differentiation observed in the tumor cells (Table 2), and the top-ranking overall hallmark changes in EMT (Figure 1J). In contrast to our data, Liu et al. identified TWIST1 and SNAI1 to be recurrently overexpressed in COSCC [10]. This discrepancy could well derive from the difference in analysis of purified tumor epithelia versus bulk tumor (containing tumor stroma), as bulk tumor sequencing has been shown to result in more “mesenchymal”, likely stroma-derived signatures [13], and TWIST1 has been shown to be expressed predominantly in the activated tumor stroma in breast cancer [31]. Even more support for a strong EMT-related reprogramming comes from the leading edge down-regulated genes, the majority of which are involved in processes associated with epithelial cell and tissue differentiation and development, and metabolic processes (Figure 2E). *Equally*, the decrease in metabolic processes is in line with a more mesenchymal phenotype that has been demonstrated to be more motile but less metabolically active than typical epithelial cells [13,21]. Finally, there is a significant correlation between EMT and expression of PD-L1 (CD274) and CTLA-4 (CD152), 2 of the key molecules that block antitumor immune response, in HNSCC [22]. Accordingly, our RNAseq dataset also revealed a significant increase in both of these inhibitory molecules in the tumor epithelium. This indicates that in COSCC, similar to human tumors, there is increased expression of PD-L1 and CTLA-4 that correlates with expression of EMT markers, suggesting that patients with COSCC could benefit from immune checkpoint inhibitor therapies, in a manner comparable to results from currently ongoing trials with HNSCC [23]. It is important to note that the TCGA dataset is built on bulk tumor sequencing, which leads to the presence of stroma-derived data in contrast to our LCM approach. As such, this could potentially lead to an overestimation of mesenchymal-like gene signatures in the TCGA data. Given the comparison with canine epithelial data, this is not expected to influence the comparative expression changes presented herein.

HNSCC pathogenesis is strongly associated with loss of TP53 function, caused either by direct mutation of TP53 through carcinogens (most prominently tobacco smoke or alcohol consumption), or through infection with human papillomavirus (HPV), whereby viral protein products bind to and inactivate TP53 [1]. Of note, an association between tumors of the nasal cavity in dogs and environmental exposure to tobacco smoke has been reported, suggesting that the occurrence of tumors in pet dogs could potentially act as “sentinel event” for human cancer risk [32]. To date, however, it remains unclear whether and to what extent development of COSCC is associated with exposure to such harmful environmental agents. HPV infection is implicated in development of more than 50% of oropharyngeal HNSCC, usually with high levels of HPV detectable in affected tumors [1], [2], [3]. The contribution of papillomaviruses (PV) to COSCC is much less clear [11]. Despite few early reports indicating the presence of PV in a limited number of COSCC [33], normally the levels of canine PV detected in COSCC are very low and only present in very few samples [10], or not at all [34]. In our analyses of 10 cases of COSCC, we did not find any indication of PV presence in the RNAseq data whatsoever (data not shown), supporting previous reports arguing against a role of PV as a major contributor to development of COSCC [35]. The involvement of TP53 in the development of COSCC is yet under debate. Nuclear overexpression of TP53 has been detected in 69% and 35% of the examined oral and nonoral canine squamous cell carcinomas [33,36]. Only very few studies have analyzed the prevalence of TP53 mutations in COSCC, and these only found very few mutations in TP53, suggesting that mutations of this gene might contribute less prominently to COSCC [10]. As such, due to the small number of analyzed cases, it remains unclear to what extent mutations in TP53 contribute to the biology of COSCC. It is interesting to note, however, that TP53 has been found to directly repress transcription of KRT14 and KRT17, both of which are heavily up-regulated in COSCC as discussed above, suggesting an impairment of TP53 activity in COSCC [37,38]. Despite some of these differences between COSCC and HNSCC, transcriptional reprogramming is highly comparable in the 2 species, with the E2F-CDK4/6 axis emerging as a salient feature. In HNSCC, components of the CyclinD – CDK4/6 pathway are often altered through various different mechanisms [1], [2], [3]. Indeed, genomic amplification of CDK6 has been detected in COSCC [10], providing a rationale for the high CDK6 levels that can be detected in these tumors. This is in line with the increased expression of genes related to cell cycle progression, such as CDK6 and E2F targets in our analysis, suggesting strong contributions from this pathway to COSCC. Along this line, it has been recently demonstrated that CDK6 antagonizes functions of TP53 during tumorigenesis, allowing for immortalization and outgrowth of primary transformed cells [39]. Hence it is tempting to speculate that while the initial molecular trigger for tumor formation might differ between the 2 species, both COSCC and HNSCC mechanistically depend on the activation of the CDK6-E2F pathway as a trigger to advance the cell cycle, as well as on inactivation of TP53 to undergo full transformation. In HNSCC, CDK6 overactivation can be found in 8% of HPV-negative cases, but not in HPV positive cases [1]. Also, in HPV-negative HNSCC, the CDK6-inhibitory factors CDKN2A and let-7c are inactivated in 57% and 40% of all cases, while no or little inactivation occurs in HPV positive tumors. The reasons for the frequent amplification and/or overexpression of CDK6 in COSCC should be further analyzed.

With respect to therapeutic vulnerabilities, activation of the CyclinD – CDK4/6 pathway can sensitize tumor cells to CDK4/6 inhibitors in HNSCC. Indeed, when combined with cetuximab, the CDK4/6 inhibitor palbociclib has shown clinical efficacy in HNSCC in a phase I trial [40] and a multicenter phase II trial [41]. Thus, CDK4/6 inhibition is currently considered as a viable option for treatment of HPV-unrelated HNSCC in humans [42,43]. In line with these findings, several clinical trials aiming to analyzing the combination of palbociclib in combination with carboplatin or PI3K/mTOR inhibitors in advanced, recurrent or unresectable cases of HNSCC and other solid tumors are in progress (clinicaltrials.gov: NCT03194373, NCT03065062). Our results strongly support the assumption that COSCC heavily rely on CDK4/6-CyclinD activation, as evidenced by significant overexpression of CDK4/6 in tumors compared to normal epithelium as well as the strong sensitivity of COSCC cell lines to palbociclib treatment in vitro (Figure 3). This suggests that treatment with palbociclib (or potentially other clinically approved CDK4/6 inhibitors) could indeed provide a valuable therapeutic option for COSCC, especially in cases where surgical resection is difficult.

## Material and methods

### Canine cases included in the study

The cases included were FFPE diagnostic specimens selected from the records of the Swiss Canine Cancer Registry (see also Table 1 for a list of all cases) [44]. They were selected based on the requirement that a sufficient amount of non-neoplastic epithelium was available on the blocks. The tumors consisted of 10 oral, nontonsillar, conventional squamous cell carcinomas [45]. One tumor was well-differentiated, eight tumors were moderately differentiated, and one was poorly differentiated.

### Laser-capture microdissection and RNA isolation

Laser-capture microdissection was performed as described in [14]. Areas for isolation were defined by a nationally-certified veterinary pathologist, and comprised on one side neoplastic epithelial tissues including all levels of differentiation present and excluding areas with obvious clusters of intraepithelial leukocytes (mainly consisting of neutrophils); on the other side they comprised adjacent, non-neoplastic epithelium. RNA from LCM-isolates was extracted as previously described [15]. RNA abundance and quality were analyzed using the 4200 Tape Station Software using the High Sensitivity RNA ScreenTape kit (Agilent Technologies), as detailed in Supplementary Table 4.

### Quantitative RT-PCR

Reverse transcription was performed using the iScript cDNA Synthesis Kit (BioRad) according to the manufacturer's protocol, using a total of 5 ng of RNA per reaction, and cDNA was preamplified using the TaqMan PreAmp Master Mix (2 ×) (Applied BiosystemsTM) according to the manufacturer's protocol using 14 PCR cycles. RT-qPCR was performed using KAPA PROBE FAST qPCR Kit Master Mix (2 ×) Universal reagents (Kapa Biosystems), with 2,5 µL cDNA/reaction in a total volume of 10 µL and reactions were run in duplicates on the CFX384 Touch Real-Time PCR detection system (BioRad). The primer details can be found in Supplementary Table 5. Quantification of gene expression was performed using the comparative CT method and values were normalized against GAPDH, PPIA and B2M as endogenous controls. Results were expressed as fold change in mRNA levels of tumor compared to normal epithelium. Primers were customized Taqman gene expression assays specifically designed to detect the canine isoforms of the targeted genes (ThermoFisher Scientific), used at final concentrations of 900 nM primers and 250 nM probes, or, for canine GAPDH, purchased from Microsynth (Balgach, Switzerland) and used at a final concentration of 300 nM primers and 200 nM probe. All primer pairs have been validated and displayed approximately 100% amplification efficiency.

### RNA sequencing

RNA sequencing was performed as previously described [15]. Briefly, RNA library preparation and depletion of ribosomal RNA was performed using the SMARTer Stranded Total RNA-seq Kit Pico Input Mammalian (Clontech) with 4 ng input RNA. The libraries were loaded onto an Illumina HiSeq5000v4 instrument and subjected to 2 × 126 cycles of paired-end sequencing according to standard protocols used at the Functional Genomics Centre Zurich (FGCZ). The raw sequencing data have been deposited in the European Nucleotide Archive with the primary accession code PRJEB34234.

### Bioinformatics analyses

The quality of RNAseq reads was assessed with FastQC (http://www.bioinformatics.babraham.ac.uk/projects/fastqc). Reads were trimmed with Trimmomatic [46] (v0.33, 4 bases hard-trimming from the start, and adaptor trimming at the end). Trimmed reads were aligned to the reference genome and transcriptome (FASTA and GTF files, Ensembl release88, CanFam3.1) with STAR [47] version 2.5.1b. Gene expression was quantified using the R/Bioconductor package R subread (version 1.24.1) [48]. Genes with consistently low counts were filtered out by keeping those with Count Per Million (CPM) value above 5.6 in at least 10 libraries. These cutoffs were set based on the sequencing depths and experimental design. CPM values were computed by “cpm” function from edgeR Bioconductor package (version 3.24.0) [48].

Raw counts were subsequently normalized and adjusted for mean-variance trend using the “rlog” function from DESeq2 Bioconductor package (version 1.22.0) [49]. Normalized data were then used to generate multidimensional scaling plot of distances between gene expression profiles with “plotMDS” function from limma Bioconductor package (version 3.38.1) [50]. Differential expression analysis was performed using DESeq2 Bioconductor package, setting padj=0.05 and log2FoldChange=1 as significance threshold [49]. Shrunken log2foldchange estimates were obtained using the original DESeq2 shrinkage estimator. Gene annotation (i.e., canine ensembl id to canine gene symbol and canine ensembl id to human ortholog mappings) were obtained using biomaRt Bioconductor package (version 2.38.0) [51]. GSEA analysis was performed with fgsea Bioconductor package (version 1.8.0) [52] with limma's paired-sample t-statistic computed on rlog-normalized data as gene ranking metric, and Hallmark gene sets obtained from MSigDB database [53]. Prior to GSEA, canine ensembl ids were mapped to human orthologs using biomaRt. If a human ortholog was associated with more than one canine ensembl id, the ensembl id with maximum variance was selected using the collapseRows function from WGCNA R package (version 1.66) [54].

Paired tumor/normal RNAseq data from TCGA's HNSCC subset (*N* = 43) was obtained from Gene Expression Omnibus under GSE62944 [55], and differential expression analysis was performed with limma Bioconductor package using the voom approach. Setting adj.P.Val = 0.01 and logFC = 1 as significance threshold, we obtained a HNSCC gene signature comprised of significantly up- or down-regulated genes in human tumors compared to their adjacent normal tissue. We next adapted 2 complementary gene set testing methods to assess the enrichment of HNSCC gene signature in COSCC samples. First, we ranked all genes in COSCC data based on their fold change expression in tumor vs normal samples. We then looked at the enrichment of HNSCC gene signature on this ranked list, using GSEA-like running-sum statistic as implemented in fgsea Bioconductor package. As the second approach, we used QuSAGE (version 2.16.0) [56], which quantifies activity of HNSCC gene signatures in COSCC data with a complete probability density function while accounting for inherent gene-gene correlations in the data. We used rlog-normalized COSCC counts summarized at the human ortholog level as input for both gene set testing methods. Moreover, since both GSEA and QuSAGE test for coordinated expression pattern in the data, we examined significantly up- and down-regulated HNSCC genes separately. Finally, to further assess the biological relevance of genes demonstrating high degree of directional homology between the 2 species, we tested for over-representation of Gene Ontology terms representing biological processes (GO-bp) among GSEA's leading-edge subsets. Over-representation analysis was performed using the GSEA webtool (https://www.gsea-msigdb.org). Over-represented GO terms are ranked based on hypergeometric *P*-value after correction for multiple hypothesis testing according to Benjamini and Hochberg.

Upstream regulatory kinases potentially responsible for the observed expression pattern in COSCC data were identified using Expression2Kinases (X2K) software [24].

Palbociclib target gene/protein list was obtained from the Comparative Toxicogenomics Database (CTD; http://ctdbase.org). GSEA analysis of palbociclib target list was performed similar to Hallmark gene sets described above.

### Cell culture

SCC1 cells were derived from a canine oral squamous cell carcinoma and were kindly donated by Prof. E. Müller [26]. CoSCC were isolated from a gingival squamous cell carcinoma of a 4 years old male Beauceron and were a kind gift of Dr. M. Wergin (Division of Radiation Oncology, Vetsuisse Faculty, University of Zurich). DUS cells were a kind gift of Prof. M. Kowalewski (Veterinary Anatomy, Vetsuisse Faculty, University of Zurich, [27]). HeLa and MDA-MB-231 cells were purchased from ATCC. Cells were cultured under standard conditions @ 37 °C in humid atmosphere with 5% CO_2_ in DMEM high glucose (Sigma) containing 15% FCS (Gibco), MEM-Non essential amino acids (Gibco) and antibiotic-antimycotic supplement (Gibco).

### Cell treatment and Resazurin assay

Twenty-four hours before treatment, 1,000 to 2,500 cells were seeded in 100 µL complete medium into white-walled 96 well plates. The stock solution of palbociclib (10 mM in DMSO; PD0332991 isethionate, Sigma) was serially diluted in complete medium to obtain the required concentrations and used to replace the seeding medium. After 72 h, 20 µL of a stock solution of 0.15 mg/mL Resazurin diluted in PBS was added into every well. Sample fluorescence was measured after 2- to 4-h incubations using the fluorospectrometer LS-55 from Perkin Elmer set to ex = 560 and em = 590. Mean values of 4 to 8 replicate wells were calculated for each treatment point and cell line, and normalized to DMSO treated control cells.

### Western blot

Whole cell extracts for Western blotting were prepared as described previously [57]. Proteins were separated on 4% to 20% Tris-Glycine gels (Novex) and transferred onto Immobilon-FL Polyvinylidene fluoride (PVDF) membranes (Millipore) according to standard procedures (Novex). Blots were probed with following antibodies: CDK6 (Novusbio, NBP1–87,262), CDK4 (Santa Cruz, sc23896), Cyclin D1 (Santa Cruz, sc8396), and α-Tubulin (Sigma, T5168). Secondary antibodies conjugated with Alexa Fluor 680 (Thermo Scientific) and IRDye 800CW (Li-cor Biosciences) fluorescent dyes were used. Detection and quantification were carried out using an Odyssey image analysis system (Li-cor Biosciences).

### Cell cycle analysis

For cell cycle analysis by FACS, trypsinised cells were fixed in ice-cold 70% ethanol for at least 30 min @−20 °C. To remove the fixation solution, cells were spun 5 min @ 250 rcf @4 °C, and the supernatant was discarded. Cells were then resuspended in phosphate buffered saline with 100 µg/mL of DNase free RNase A (Sigma) and incubated @37 °C for 30 min, and further stained with 10 µg/mL propidium iodide (Sigma). Samples were run on a Fortessa (BD Biosciences) and the cell cycle distribution analyzed using FlowJo V10.6.1.

### Analysis of apoptosis and necrosis

Analysis of apoptotic and necrotic cells was performed with the Annexin V-FITC Apoptosis Staining/Detection Kit (Abcam, ab14085) according to the manufacturer's protocol. Annexin V is used to label phosphatidylserine sites on the membrane surface of apoptotic cells. Propidium iodide (PI) is used to label the cellular DNA in necrotic cells where the cell membrane has been totally compromised. This combination allows the differentiation among early apoptotic cells (annexin V positive, PI negative), late apoptotic cells (annexin V positive, PI positive), necrotic cells (annexin V negative, PI positive), and viable cells (annexin V negative, PI negative). Briefly, cells treated with 0.1 µM palbociclib for 72 h were washed and adherent cells trypsinised. Trypsin was neutralized using serum containing medium, 500,000 cells were collected by centrifugation, and resuspended in 500 µL 1X Binding Buffer. 5 µL Annexin V-FITC and 5 µL Propidium Iodide were added, and samples were incubated at room temperature for 5 min before acquisition with FACS as detailed above.

### Statistical analysis and graphical display of results

All statistical analysis, calculation and graphical display was performed with the program GraphPad Prism (www.graphpad.com). Statistical significance of gene expression changes detected by RNAseq were calculated using a generalized linear model with the patient as secondary factor, i.e., paired tests.

## Author contributions

FG was co-responsible for study design, supervision and funding, and, as a nationally certified veterinary pathologist, performed assessment and selection of clinical cases, defined selection of areas of interest by LCM and data analysis. SN was responsible for data processing and bioinformatics analyses. EB performed cell sensitivity assays, FACS and apoptosis analyses and qRT-PCR. IRB performed LCM. RG contributed to data analysis and to the first draft of the manuscript. EM was co-responsible for study design, supervision and funding, and responsible for isolation of RNA, qRT-PCR, Western Blot, analyses involving cell culture and data analysis. EM wrote the first draft of the manuscript. All authors read, contributed to, and approved the final manuscript.

## Availability of data and materials

The sequence data of this study have been deposited in the European Nucleotide Archive with the primary accession code PRJEB34234. All other data supporting our findings is contained in the manuscript and in the additional files.

## Appendix Supplementary materials

Image, application 1

Image, application 2

Image, application 3

Image, application 4
