# Supplementary material for: Molecular homology between canine spontaneous oral squamous cell carcinomas and human head-and-neck squamous cell carcinomas reveals disease drivers and therapeutic vulnerabilities
Source: Neoplasia. 2020 Nov 2;22(12):778–88. doi: 10.1016/j.neo.2020.10.003 (PMC7642746; doi:10.1016/j.neo.2020.10.003)
Supplement: Supplementary file 1 [file mmc1.docx]

Guscetti *et al,* 2020

Supplementary Figures and Tables

**Supplementary Figure 1:** A-C) Histological appearance of COSCC (example case no. 6). A) Overview of infiltratively growing, moderately differentiated canine oral squamous cell carcinoma. B) Detail of invasive front of the tumour. C) Detail of adjacent non-neoplastic epithelium. D) Heatmap of expression values for 340 significantly up- and 329 significantly down-regulated genes, as shown in Figure 1B. Significance was determined using |FC|>2 and FDR<0.05 as cutoff values. Labels below the columns indicate tumour and normal samples and the case number, respectively.

**Supplementary Figure 2:** A) Overview of human samples from TCGA GSE62944 by anatomical site. B) Principal component analysis (PCA) of tumour and matched normal samples of HNSCC from TCGA GSE62944. PCA was performed using all genes.

**
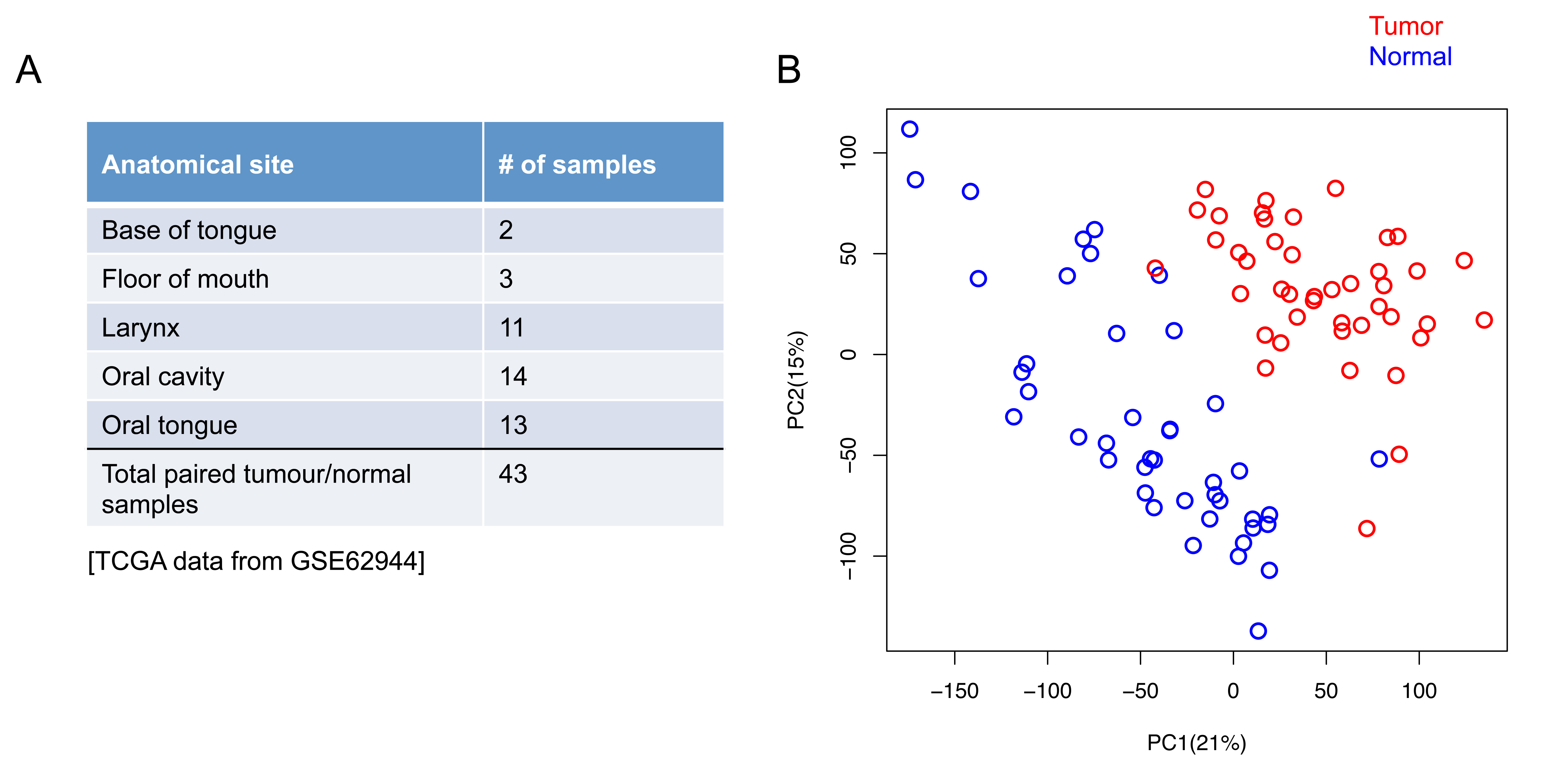
**

**Supplementary Table 4.** Summary of RNA concentration, yield and quality of samples used in this study.

| Case # | Sample | Concentration  (pg/ul) | Total yield  (ng) | DV200 (%) |
| --- | --- | --- | --- | --- |
| 1 | Normal | 5050 | 160 | 73.99 |
|  | Tumour | 603 | 25 | 67.74 |
| 2 | Normal | 1930 | 85 | 55.52 |
|  | Tumour | 1500 | 54 | 67.25 |
| 3 | Normal | 773 | 23 | 68.96 |
|  | Tumour | 1480 | 77 | 73.14 |
| 4 | Normal | 2130 | 64 | 59.95 |
|  | Tumour | 3810 | 192 | 61.11 |
| 5 | Normal | 1460 | 65 | 72.02 |
|  | Tumour | 1170 | 55 | 74.10 |
| 6 | Normal | 2530 | 100 | 74.46 |
|  | Tumour | 816 | 40 | 71.62 |
| 7 | Normal | 1180 | 77 | 71.83 |
|  | Tumour | 1010 | 45 | 71.54 |
| 8 | Normal | 1990 | 70 | 48.33 |
|  | Tumour | 1340 | 49 | 53.73 |
| 9 | Normal | 1150 | 42 | 66.51 |
|  | Tumour | 1180 | 45 | 66.21 |
| 10 | Normal | 3870 | 19 | 35.81 |
|  | Tumour | 1460 | 43.8 | 34.19 |

**Supplementary Table 5**. List of primers used for qRT-PCR. The “c” before each gene indicates that primers were designed to detect the canine isoforms of the intended targets.

| Gene target | Sequence | Amplicon length | Assay ID/Ref |  |
| --- | --- | --- | --- | --- |
| cCDK6 | Manufacturer’s proprietary information | 75 nt | AR7DRNZ |  |
| cPDL1/CD274 | Manufacturer’s proprietary information | 75 nt | Cf04947241_m1 |  |
| cZEB2 | Manufacturer’s proprietary information | 81 nt | Cf02708878_m1 |  |
| cFN1 | Manufacturer’s proprietary information | 56 nt | Cf00415008_m1 |  |
| cCDK4 | Manufacturer’s proprietary information | 64 nt | Cf04330666_g1 |  |
| cGAPDH | \| Fw: 5’-GCTGCCAAATATGACGACATCA-3’ \| \| --- \| \| Re: 5’-GTAGCCCAGGATGCCTTTGAG-3’ \| \| Probe: 5’-TCCCTCCGATGCCTGCTTCACTACCTT-3’ \| | 75 nt | [1] |  |
| cB2M | Manufacturer’s proprietary information | 87 nt | Cf02659077_m1 |  |
| cPPIA | Manufacturer’s proprietary information | 92 nt | Cf03986523_gH |  |

**References**

1. Ettlin J, Clementi E, Amini P, et al (2017) Analysis of Gene Expression Signatures in Cancer-Associated Stroma from Canine Mammary Tumours Reveals Molecular Homology to Human Breast Carcinomas. Int J Mol Sci 1–19. doi: 10.3390/ijms18051101
